# Identification of subgroups of patients with type 2 diabetes with differences in renal function preservation, comparing patients receiving sodium‐glucose co‐transporter‐2 inhibitors with those receiving dipeptidyl peptidase‐4 inhibitors, using a supervised machine‐learning algorithm (PROFILE study): A retrospective analysis of a Japanese commercial medical database

**DOI:** 10.1111/dom.13753

**Published:** 2019-06-03

**Authors:** Fang L. Zhou, Hirotaka Watada, Yuki Tajima, Mathilde Berthelot, Dian Kang, Cyril Esnault, Yujin Shuto, Hiroshi Maegawa, Daisuke Koya

**Affiliations:** ^1^ Real World Evidence Generation, Sanofi Bridgewater New Jersey; ^2^ Department of Metabolism and Endocrinology, Juntendo University Graduate School of Medicine Tokyo Japan; ^3^ Medical Affairs, Sanofi K.K. Tokyo Japan; ^4^ Data Science Consulting, Quinten Paris France; ^5^ Department of Medicine, Shiga University of Medical Science Otsu Japan; ^6^ Department of Diabetology and Endocrinology, Kanazawa Medical University Uchinada Japan

**Keywords:** DPP‐4 inhibitor, machine‐learning algorithm, real‐world clinical practice, renal function, SGLT2 inhibitor, type 2 diabetes

## Abstract

**Aims:**

To investigate the effects of sodium‐glucose co‐transporter‐2 (SGLT2) inhibitors vs. dipeptidyl peptidase‐4 (DPP‐4) inhibitors on renal function preservation (RFP) using real‐world data of patients with type 2 diabetes in Japan, and to identify which subgroups of patients obtained greater RFP benefits with SGLT2 inhibitors vs. DPP‐4 inhibitors.

**Methods:**

We retrospectively analysed claims data recorded in the Medical Data Vision database in Japan of patients with type 2 diabetes (aged ≥18 years) prescribed any SGLT2 inhibitor or any DPP‐4 inhibitor between May 2014 and September 2016 (identification period), in whom estimated glomerular filtration rate (eGFR) was measured at least twice (baseline, up to 6 months before the index date; follow‐up, 9 to 15 months after the index date) with continuous treatment until the follow‐up eGFR. The endpoint was the percentage of patients with RFP, defined as no change or an increase in eGFR from baseline to follow‐up. A proprietary supervised learning algorithm (Q‐Finder; Quinten, Paris, France) was used to identify the profiles of patients with an additional RFP benefit of SGLT2 inhibitors vs. DPP‐4 inhibitors.

**Results:**

Data were available for 990 patients prescribed SGLT2 inhibitors and 4257 prescribed DPP‐4 inhibitors. The proportion of patients with RFP was significantly greater in the SGLT2 inhibitor group (odds ratio 1.27; *P* = 0.01). The Q‐Finder algorithm identified four clinically relevant subgroups showing superior RFP with SGLT2 inhibitors (*P* < 0.1): no hyperlipidaemia and eGFR ≥79 mL/min/1.73 m^2^; eGFR ≥79 mL/min/1.73 m^2^ and diabetes duration ≤1.2 years; eGFR ≥75 mL/min/1.73 m^2^ and use of antithrombotic agents; and haemoglobin ≤13.4 g/dL and LDL cholesterol ≥95.1 mg/dL. In each profile, glycaemic control was similar in the two groups.

**Conclusion:**

SGLT2 inhibitors were associated with more favourable RFP vs. DPP‐4 inhibitors in patients with certain profiles in real‐world settings in Japan.

## 1 INTRODUCTION

Sodium‐glucose co‐transporter‐2 (SGLT2) inhibitors suppress glucose reabsorption in the kidney to increase urinary glucose excretion, resulting in reductions in fasting and postprandial glucose. These improvements are coupled with changes in adiposity, substrate utilization, lipolysis, hormone secretion and central regulation of appetite.^1^, ^2^ SGLT2 inhibitors also appear to have beneficial effects with regard to renal function. For example, in the EMPA‐REG OUTCOME trial of patients with type 2 diabetes and high risk of cardiovascular events, significantly fewer patients in the empagliflozin group experienced incident or worsening nephropathy, or a doubling of the serum creatinine level accompanied by an estimated glomerular filtration rate (eGFR) of ≤45 mL/min/1.73 m^2^ vs. placebo.^3^ A subsequent analysis indicated that empagliflozin was associated with a reduction in intraglomerular pressure, which possibly contributed to the preservation of eGFR during chronic therapy.^4^ Similar findings were also reported in the CANVAS study programme, which showed that canagliflozin was associated with an attenuated eGFR decline and a reduction in albuminuria compared with placebo.^5^ These effects of canagliflozin were not modified by the baseline kidney function or history/high risk of cardiovascular disease in patients with eGFR ≥30 mL/min/1.73 m^2^ at baseline.^6^ These results were reiterated in a recent meta‐analysis of 40 randomized controlled trials comprising 29 954 participants, which showed that SGLT2 inhibitors preserve renal function in patients with diabetes with or without renal impairment.^7^

Six SGLT2 inhibitors (canagliflozin, dapagliflozin, empagliflozin, ipragliflozin, luseogliflozin and tofogliflozin) have been available for the treatment of type 2 diabetes for ~5 years in Japan. These drugs showed good efficacy and safety as monotherapy or in combination with alternative oral antidiabetic drugs or insulin in clinical trials, and post‐marketing surveillance studies.^8^, ^9^, ^10^, ^11^, ^12^, ^13^, ^14^, ^15^, ^16^, ^17^, ^18^, ^19^, ^20^, ^21^, ^22^ However, there is limited information regarding whether SGLT2 inhibitors help preserve renal function in Japanese patients in clinical practice. The aim of the present study, therefore, was to investigate whether SGLT2 inhibitors preserve renal function in Japanese patients with type 2 diabetes in real‐world clinical practice. For this purpose, we analysed a large Japanese medical database using patients treated with dipeptidyl peptidase‐4 (DPP‐4) inhibitors as a reference group because these drugs have been available in Japan for ~8 years, have a complementary mechanism of action to SGLT2 inhibitors and, in our experience, are widely prescribed to patients with type 2 diabetes in Japan. Indeed, a recent analysis of two Japanese clinical databases (Japan Medical Data Centre [JMDC] database and Medical Data Vision [MDV] database) showed that DPP‐4 inhibitors were frequently prescribed in previously untreated patients (JMDC: 44.0%; MDV: 54.8%) and as part of combination therapy in previously treated patients (JMDC: 74.6%; MDV: 81.1%).^23^ Adherence and persistence to DPP‐4 inhibitors were also found to be high in both databases. Although prior studies have indirectly compared the effects of SGLT2 inhibitors and DPP‐4 inhibitors on glycaemic control, body weight and cardiovascular events,^24^, ^25^ none, to our knowledge, has compared their effects on renal function. Furthermore, the effects of DPP‐4 inhibitors on renal function in clinical practice are not well established.^26^, ^27^ The present study, therefore, offered us an opportunity to explore the effects of SGLT2 inhibitors and DPP‐4 inhibitors on renal function in patients with type 2 diabetes.

Our primary objective was to identify which subgroups of patients may have better renal function preservation (RFP) with SGLT2 inhibitors than with DPP‐4 inhibitors. Given the heterogeneity of patients with type 2 diabetes, it is likely that certain subgroups of patients may show better responses to SGLT2 inhibitors than to DPP‐4 inhibitors; such information may help clinicians determine which class of drug might be more suitable for specific patients. We used a machine‐learning algorithm^28^ to identify the profiles of patients who were more likely to experience RFP with an SGLT2 inhibitor than with a DPP‐4 inhibitor. For each of the profiles, we also evaluated whether the change in glycated haemoglobin (HbA1c) with an SGLT2 inhibitor was superior or equivalent to that achieved with a DPP‐4 inhibitor.

To achieve these objectives, we extracted data from the Japanese MDV database, which has recorded health claims data and administrative data or diagnosis procedure combination data for over 20 million patients treated at over 300 acute care hospitals in Japan since April 2010. We identified patients treated with either an SGLT2 inhibitor or a DPP‐4 inhibitor for at least 9 months whose renal function was measured after starting treatment.

## 2 METHODS

### 2.1 Ethics

As this was a retrospective study of anonymous data from an administrative database, ethical approval was not necessary.

### 2.2 Data source

For the present study, we used the Japanese MDV database. Since April 2010, this database has collected health claims, administrative, and diagnosis procedure combination data for >20 million patients at >300 Japanese acute hospitals (20% of all acute care hospitals in Japan). The hospitals directly record data in the database. All of the hospitals participate in the diagnosis procedure combination/per‐diem payment system in Japan, and the database broadly reflects the population of Japan in terms of age and gender distribution. The database does not show geographical bias because the participating hospitals are distributed throughout Japan.

### 2.3 Study population

The following data were recorded in the database: age; sex; date of diagnosis; disease code; International Classification of Diseases 10th revision (ICD‐10) code; drug name/class; date of prescription; medical procedure; date of visit; and laboratory test results. Using this database, we identified all patients aged ≥18 years with a diagnosis of type 2 diabetes mellitus (ICD‐10 codes in Table S1), who had at least one prescription of any SGLT2 inhibitor or any DPP‐4 inhibitor in the identification period (May 1, 2014 to September 30, 2016), and had at least two valid creatinine values between 0.2 and 20 mg/dL taken 6 months before the index date (baseline) and 9 to 15 months after the index date (follow‐up). Patients with at least one diagnosis code for type 1 diabetes mellitus were excluded. The index date was defined as the first prescription claim of a SGLT2 inhibitor or DPP‐4 inhibitor in the identification period. We only included patients who were active in the database for ≥6 months prior to the index date.

**Figure 1:**
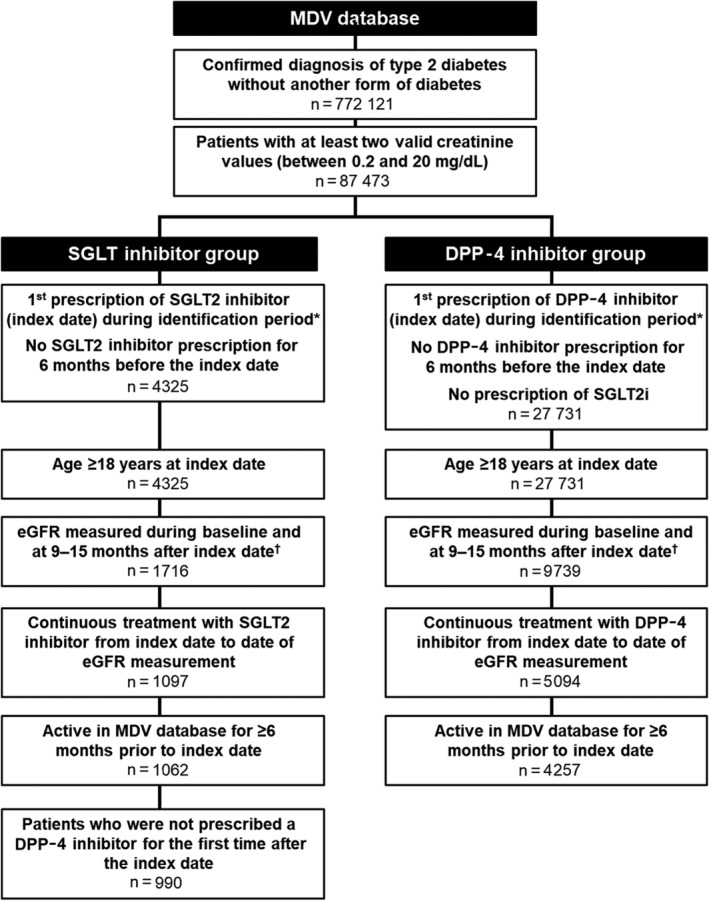
Patient selection. *Identification period was May 1, 2014 to September 30, 2016. ^†^eGFR was to have been measured at least once during baseline period and at least once between 9 and 15 months after index date. DPP‐4, dipeptidyl peptidase‐4; eGFR, estimated glomerular filtration rate; MDV, Medical Data Vision; SGLT2, sodium‐glucose co‐transporter‐2

Patients were included in the SGLT2 inhibitor group or the DPP‐4 inhibitor group if they had used any SGLT2 inhibitor or any DPP‐4 inhibitor continuously for at least 9 months starting from the index date. The SGLT2 group excluded any patients who were newly prescribed DPP‐4 inhibitors for the first time after the index date. The DPP‐4 inhibitor group excluded any patients prescribed SGLT2 inhibitors. Patients were excluded if they lacked data in the MDV database for at least 6 months prior to the index date. The patient selection criteria are summarized in Figure 1. Further eligibility criteria are summarized in the Appendix S1.

### 2.4 Study outcomes

The index date was the date on which the SGLT2 inhibitor or DPP‐4 inhibitor was first prescribed. Baseline was defined as the 6‐month period prior to the index date. Patients were followed for 9 to 15 months from the index date (follow‐up period).

Renal function was assessed in terms of the eGFR, calculated using the Japanese equation for eGFR^29^:

eGFR = 194 × creatinine^−1.094^ × age^−0.287^(×0.739 for women).

The available eGFR value recorded in the follow‐up period was analysed; for patients with multiple eGFR values during follow‐up, the value closest to 12 months was used. The study outcome, RFP, was defined as no change or an increase in the eGFR from the baseline value (last eGFR value recorded before the index date) to the value in the follow‐up period. A decrease in eGFR from the baseline value to the follow‐up value was defined as “no RFP”.

Glycaemic control was assessed in terms of changes in HbA1c from baseline (ie, last value measured before the index date) to the follow‐up value (value recorded 9‐15 months after the index date).

### 2.5 Data analyses

**Figure 2:**
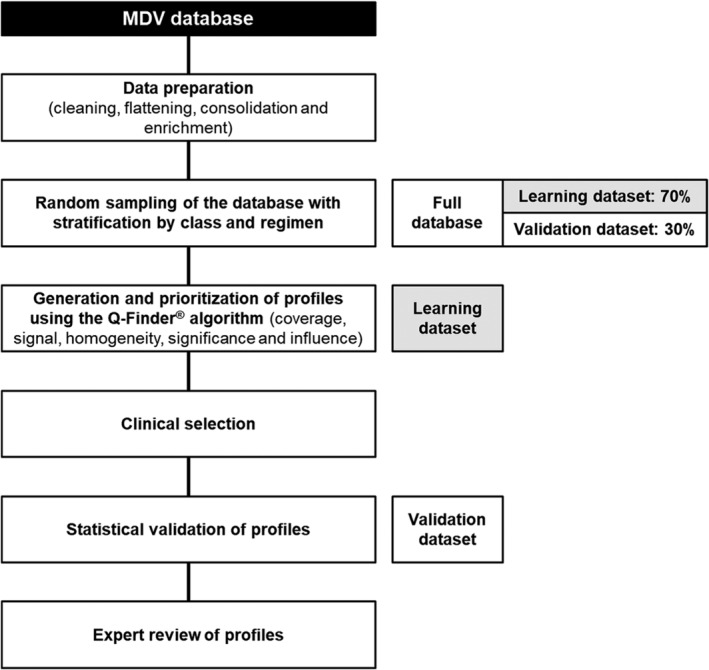
Overview of the data analysis procedure. Data preparation comprised cleaning (eg, standardization of units and dates, checking missing values), flattening/enrichment (eg, combining several rows of data into a single row for individual patients) and consolidation (merging multiple tables into a single database)

The data analytical approach is illustrated in Figure 2.

First, we compared the baseline demographics and clinical covariates between the two groups using χ^2^ tests for categorical variables and Student's *t*‐tests for continuous variables to determine *P* values. In addition to *P* values, standardized differences were calculated to distinguish practical from statistical significance.

We performed the first step of multivariable analyses to compare RFP and HbA1c between the two groups using the full study cohort by applying logistic regression (for RFP) or Gaussian regression (HbA1c) models, adjusted for propensity scores, which were based on sociodemographic and clinical covariates. The following confounding factors were used to generate generalized propensity scores for each patient included in the multivariable analyses of RFP and HbA1c: age (18‐44, 45‐64, ≥65 years); hospitalization status at baseline (inpatient, outpatient); eGFR at baseline (continuous variable); HbA1c at baseline (<6.5%, ≥6.5% to <7%, ≥7%); Charlson Comorbidity Index; gender; hyperlipidaemia; hypertension; baseline treatments (diuretics [Anatomical Therapeutic Chemical classification code C03], β blockers [C07], calcium channel blockers [C08], renin‐angiotensin system drugs [C09]), neuropathy, nephropathy, retinopathy and prior antidiabetic treatment regimen (treatment‐naïve, one oral antidiabetic drug, two or more antidiabetic drugs, insulin). Patients were not matched using the propensity scores. The confounding factors were selected based on the variables recorded in the database and our consideration of which factors were likely to confound the analysis.

Next, we used the Q‐Finder algorithm^28^ (Quinten, Paris, France) to identify the profiles of patients who experienced an additional clinical benefit using SGLT2 inhibitors over DPP‐4 inhibitors in terms of RFP. Briefly, Q‐Finder is a proprietary non‐parametric subgroup discovery algorithm that is able to detect subpopulations associated with a phenomenon of interest. It performs an exhaustive search without a *priori* hypothesis over every variable threshold combination and then performs a statistical credibility assessment for each generated subgroup through a set of chosen metrics. Thus, by only selecting the most credible subgroups, Q‐Finder is able to generate a limited set of data‐driven subgroups to test on independent data, thus preserving the statistical power while testing for robustness. The algorithm outputs a set of profiles (profile = subgroup) with higher rates of the outcome of interest (ie, in the SGLT2 inhibitor group in the present study), each profile being characterized by one or a combination of criteria. For this study, the Q‐Finder algorithm was programmed to generate profiles with a limit of two clinical criteria combinations. The profiles can be characterized by continuous variables with lower and upper bounds or modalities for qualitative variables. In addition to the statistical assessment performed by the algorithm, each profile was reviewed by a panel of experts to ensure it was clinically relevant.

Patients were randomly allocated to a learning dataset (70% of patients in the global dataset) or a validation dataset (remaining 30% of patients), stratified by treatment class. The Q‐Finder algorithm was applied to the learning dataset to generate profiles. The profiles obtained in the learning dataset were selected based on the following statistical indicators: sample size of ≥10%; homogeneity of class repartition between the profile and the learning dataset (±10%); a significantly better RFP as a “class effect” (ie, SGLT2 inhibitor effect greater than the DPP‐4 inhibitor effect within the profile with an adjusted odds ratio [aOR] ≥1.5); a significantly better RFP as a “class benefit” (SGLT2 inhibitor effect vs DPP‐4 inhibitor effect that was greater within the profile than outside the profile with a ratio of aORs of ≥1.5). To control for confounding factors, the logistic/Gaussian models for class effect and benefit included propensity scores. Next, these statistically robust profiles were reviewed by medical experts to narrow down the profiles to those considered to be clinically relevant. Finally, the top profiles from the learning dataset (ie, those that were both statistically robust and clinically relevant) were applied to the validation dataset. For validation, we examined the same statistical indicators as in the learning dataset. Moreover, to test for statistical significance, in addition to determining the *P* value as above, we also performed multiple test correction using the Benjamini–Hochberg procedure with significance defined as a risk level of <10%. Profiles that showed significance in the validation dataset were considered final profiles, and are presented in this report.

The final profiles were then applied to compare the change in HbA1c to investigate whether the change in HbA1c in the SGLT2 inhibitor group was superior to or equivalent to that in the DPP‐4 inhibitor group within each profile. Sensitivity analyses were also done in which the definition of RFP was expanded to a difference in eGFR between baseline and follow‐up of greater than −5%. This definition was applied to the four selected profiles using the learning, validation and global datasets.

## 3 RESULTS

### 3.1 Patients

Among 772 121 patients with type 2 diabetes in the MDV database in the study period, a total of 5247 satisfied the eligibility criteria, of which 990 were treated with an SGLT2 inhibitor and 4257 were treated with a DPP‐4 inhibitor (Figure 1).

Tables S2 to S6 summarize the patients' baseline characteristics, antidiabetic therapy, medical history, medical therapy and baseline laboratory variables, respectively.

There were statistically significant differences between the two groups for several baseline variables. Of particular note, the SGLT2 inhibitor group was younger (mean age 58 vs. 69 years; *P* < 0.0001), had a longer duration of diabetes (mean 7 vs. 5 years; *P* < 0.0001), were heavier (mean body weight 78 vs. 61 kg), and included a greater proportion of outpatients at baseline (93% vs. 74%; Table S2).

In terms of antidiabetic therapy (Table S3), a greater proportion of patients in the SGLT2 inhibitor group were on two or more oral antidiabetic drugs at baseline (53% vs. 10%), with a mean of 2.2 vs. 0.5 oral antidiabetic drugs (*P* < 0.0001), with significant differences in the prior use of biguanides (64% vs. 16%; *P* < 0.0001), and sulphonylureas (35% vs. 15%) in particular. Overall, 72% of the patients in the SGLT2 inhibitor group used a DPP‐4 inhibitor at baseline.

Patients in the SGLT2 inhibitor group tended to have more comorbidities, with a significantly higher Charlson Comorbidity Index (2.1 vs. 1.8; *P* < 0.0001) and higher rates of acute myocardial infarction (9% vs. 5%), congestive heart failure (21% vs. 14%), dyslipidaemia (70% vs. 39%), hyperlipidaemia (69% vs. 39%), hypertension (71% vs. 48%), mild liver disease (23% vs. 12%), nephropathy (10% vs. 4%) and neuropathy (63% vs. 32%; all *P* < 0.0001). The rate of cancer (6% vs. 13%) was significantly lower in the SGLT2 inhibitor group (Table S4).

Consistent with the differences in comorbidities and medical history, there were some differences in the medical therapies prescribed to both groups (Table S5) and laboratory variables (Table S6). As indicated in Table S6, baseline eGFR was higher in the SGLT2 inhibitor group (76.0 vs. 65.7 mL/min/1.73 m^2^; *P* < 0.0001). Baseline HbA1c was significantly greater in the SGLT2 inhibitor group (8.3 vs. 7.7%; *P* < 0.0001) and a higher proportion of patients had HbA1c ≥7% at baseline in that group (87% vs. 63%; *P* < 0.0001). There were also significant differences in other laboratory variables, reflecting the differences in comorbidities and medical histories between the two groups.

### 3.2 Multivariable analysis of RFP and HbA1c in the full study cohort

**Figure 3:**
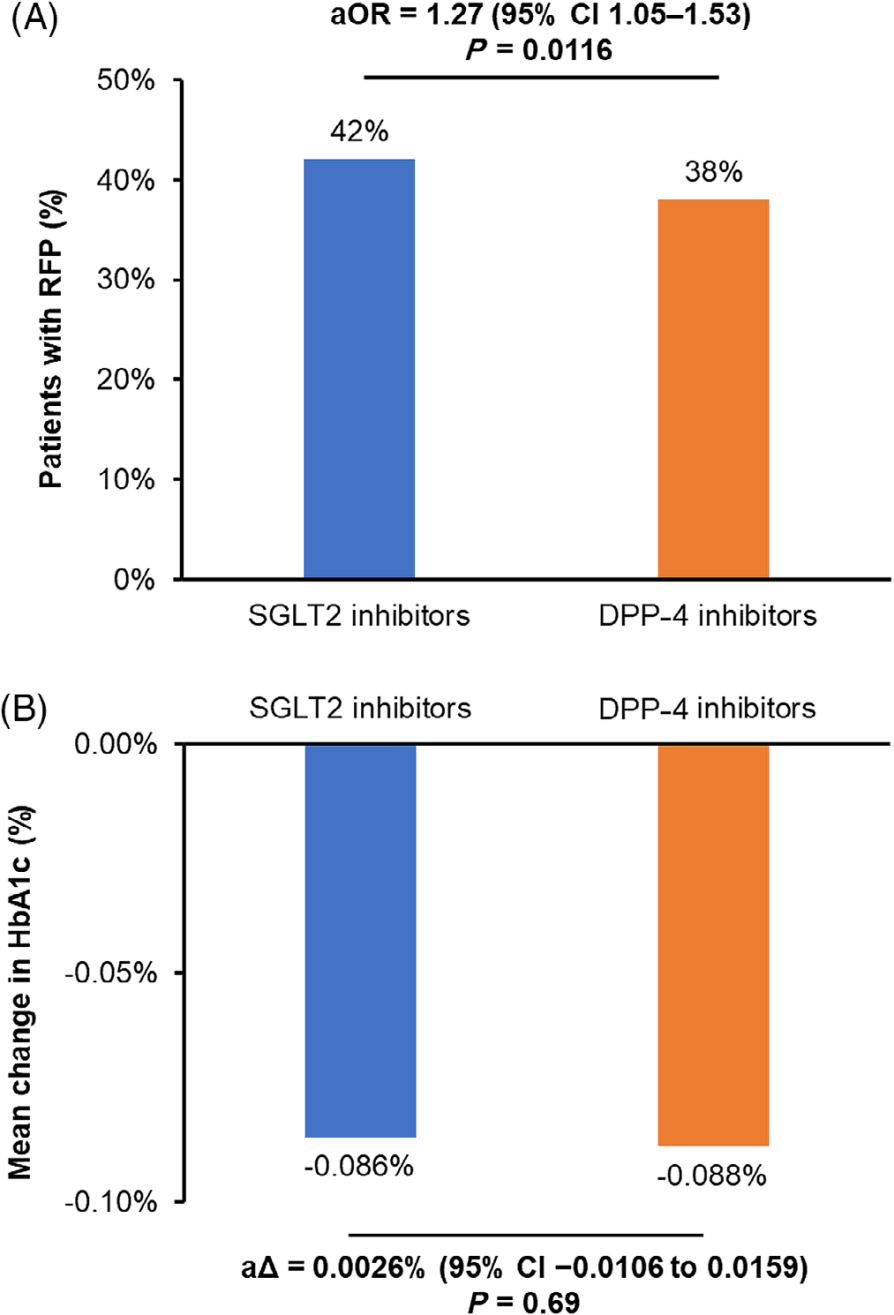
Comparison of A, percentage of patients with renal function preservation (RFP) and B, mean change in glycated haemoglobin (HbA1c) between patients treated with sodium‐glucose co‐transporter‐2 (SGLT2) inhibitors or dipeptidyl peptidase‐4 (DPP‐4) inhibitors in the overall database. RFP was defined as an increase or no change in estimated glomerular filtration rate between the baseline and follow‐up values. The adjusted mean difference in HbA1c was calculated as the between‐group difference in the mean change between the baseline and follow‐up values. aΔ, adjusted mean difference; aOR, adjusted odds ratio; CI, confidence interval.

We performed multivariable analysis in the full cohort of 5247 patients to compare the proportions of patients with RFP and the mean change in HbA1c between the SGLT2 inhibitor and DPP‐4 inhibitor groups, with adjustment for baseline confounding factors using propensity scores. As illustrated in Figure 3A, patients were more likely to achieve RFP in the SGLT2 inhibitor group than in the DPP‐4 inhibitor group (aOR 1.27, 95% confidence interval [CI] 1.05‐1.53; *P* = 0.0116, c‐statistic = 0.89). By way of comparison, the unadjusted odds ratio (OR) for RFP was 1.17 (95% CI 1.01‐1.34; *P* = 0.031). The adjusted mean change in HbA1c was nearly identical in both groups (Figure 3B).

### 3.3 Profiles for RFP

The Q‐Finder algorithm evaluated 150 profiles, of which 43 showed significant class effects and class benefits favouring SGLT2 inhibitors vs DPP‐4 inhibitors on RFP. Twelve of these profiles were deemed clinically relevant and included in profile validation. Four profiles were ultimately validated, showing similar and significant class effects and class benefits in both learning and validation datasets (Table 1). The four profiles were as follows: (A) hyperlipidaemia = no and eGFR ≥79 mL/min/1.73 m^2^; (B) eGFR ≥79 mL/min/1.73 m^2^ and duration of diabetes ≤1.2 years; (C) eGFR ≥75 mL/min/1.73 m^2^ and use of antithrombotic agents at baseline; and (D) haemoglobin ≤13.4 g/dL and LDL cholesterol ≥95.1 mg/dL. These profiles comprised 20% (n = 724), 15% (n = 556), 12% (n = 430) and 10% (n = 384) of patients in the learning dataset, respectively. Some patients were included in multiple profiles (Table S7). The class effect (aOR) ranged from 2.2 to 2.7, and class benefit (ratio of aORs) ranged from 2.0 to 2.4 in the learning dataset. Similar values were obtained when these profiles were applied to the validation dataset. The Benjamini–Hochberg *P* values were <0.1 for the class effects for all four profiles and for the class benefit for profile A in the validation dataset, validating these profiles identified using the learning dataset. The c‐statistics ranged from 0.79 to 0.82 in the learning dataset and from 0.80 to 0.84 in the validation dataset. The other eight profiles that were evaluated are listed in Table S8.

**Table 1:** Characteristics of the four profiles: Outcome = renal function preservation

Profile		Learning dataset					Validation dataset						
		N (%)	**Class effect** **(within the profile)**		Class benefit (within vs. without the profile)		N (%)	**Class effect** **(within the profile)**			**Class benefit** **(within vs. without the profile)**		
			aOR	*P* value	Ratio of aORs	*P* value		aOR	*P* value	BH*P* value	Ratio of aORs	*P* value	BH *P* value
A. Hyperlipidaemia	No	724 (20)	2.2	**0.0009**	2.1	**0.0025**	296 (19)	2.7	**0.0053**	**0.0639**	2.8	**0.0054**	**0.0646**
eGFR	≥79 mL/min/1.73 m^2^												
B. eGFR	≥79 mL/min/1.73 m^2^	556 (15)	2.7	**0.0023**	2.4	**0.0074**	238 (15)	3.0	**0.0184**	**0.0752**	2.9	**0.0293**	0.139
Duration of diabetes	≤1.2 years												
C. eGFR	≥75 mL/min/1.73 m^2^	430 (12)	2.3	**0.0032**	2.2	**0.0086**	198 (13)	2.6	**0.0212**	**0.0752**	2.5	**0.0348**	0.139
Antithrombotic agents	Yes												
D. Haemoglobin	≤13.4 g/dL	384 (10)	2.7	**0.0009**	2.0	**0.0254**	176 (11)	3.3	**0.0251**	**0.0752**	2.6	0.073	0.2189
LDL cholesterol	≥95.1 mg/dL												

*Note: *Bold font indicates statistical significance at risk levels of 5% for P values and 10% for BH P values.

Abbreviations: aOR, adjusted odds ratio; BH, Benjamini–Hochberg; eGFR, estimated glomerular filtration rate.

Sensitivity analyses using the expanded definition of RFP yielded consistent results with the primary analysis in terms of class effects and class benefits for the four selected profiles (Table S9).

### 3.4 Profiles for HbA1c change

For the profiles described in the previous section, we also considered glycaemic control in terms of the HbA1c change. There were no significant differences in the change in HbA1c between the two treatment groups in any of the four selected profiles (Table 2) or the eight additional profiles (Table S10) in either the learning or validation datasets.

**Table 2:** Characteristics of the four profiles: Outcome = HbA1c change

Profile		Learning dataset					Validation dataset						
		N (%)	Class effect (within the profile)		Class benefit (within vs. without the profile)		N (%)	Class effect (within the profile)			Class benefit (within vs. without the profile)		
			aΔ	*P* value	aΔ	*P* value		aΔ	*P* value	BH *P* value	aΔ	*P* value	BH *P* value
A. Hyperlipidaemia	No	724 (20)	−0.01	0.72	0.00	0.83	296 (19)	0.00	0.92	0.99	−0.01	0.77	0.83
eGFR	≥79 mL/min/1.73 m^2^												
B. eGFR	≥79 mL/min/1.73 m^2^	556 (15)	0.02	0.49	0.02	0.34	238 (15)	0.03	0.30	0.99	0.03	0.38	0.83
Duration of diabetes	≤1.2 years												
C. eGFR	≥75 mL/min/1.73 m^2^	430 (12)	−0.02	0.26	−0.02	0.27	198 (13)	−0.01	0.77	0.99	−0.02	0.50	0.83
Antithrombotic agents	Yes												
D. Haemoglobin	≤13.4 g/dL	384 (10)	0.01	0.79	0.01	0.78	176 (11)	0.01	0.75	0.99	0.01	0.74	0.83
LDL cholesterol	≥95.1 mg/dL												

Abbreviations: aΔ, adjusted mean difference; BH, Benjamini–Hochberg; eGFR, estimated glomerular filtration rate.

## 4 DISCUSSION

The current guidelines developed by the Japanese Diabetes Society recommend that the type(s) of glucose‐lowering agents should be individualized for each patient according to the disease characteristics as well as the pharmacological and safety profiles of each drug.^30^ In the present study, we observed marked differences in the baseline characteristics of patients treated with SGLT2 inhibitors or DPP‐4 inhibitors in real‐world settings in Japan, suggesting that SGLT2 inhibitors are often used later in the treatment regimen. We also speculate that clinicians favoured SGLT2 inhibitors in patients with hypertension and obesity in this real‐world setting, after considering the effects of SGLT2 inhibitors on lowering blood pressure and body weight reported in international studies^31^ and in Japanese studies.^32^, ^33^ Clinicians might also be delaying SGLT2 inhibitors owing to potential concern about safety.^34^ These differences in baseline characteristics should be considered when interpreting data from real‐world clinical practice.

Many patients with type 2 diabetes experience progressive renal function decline, nephropathy and chronic kidney disease. Recent studies have indicated that SGLT2 inhibitors may help to preserve renal function.^35^ We investigated whether subgroups of patients treated with SGLT2 inhibitors were more likely to show RFP in patients in real‐world clinical practice in Japan, using DPP‐4 inhibitors as a comparator. Renal function was assessed in terms of RFP (defined using eGFR). Because many patients with type 2 diabetes experience a decline in renal function over time and are at risk of progression to chronic kidney disease or end‐stage kidney disease,^35^ our observation that renal function was preserved in some subgroups of patients is clinically relevant, and suggests that SGLT2 inhibitors may delay progression to chronic kidney disease/end‐stage kidney disease. We defined RFP as no change or a positive change in eGFR over 1 year, considering the number of patients needed to show RFP in the MDV database in order to apply the analytical methods to derive the patient profiles. In the study mentioned above,^35^ a 30% or 40% decrease in eGFR over 2 or 3 years was adopted as a surrogate endpoint for progression to end‐stage renal disease in Japanese patients with chronic kidney disease. RFP in the present study should not be directly compared with renal protection in that study owing to the different definitions of renal outcome and different follow‐up periods. The proportion of patients who maintained eGFR at 1 year in the SGLT2 inhibitor group was 42%, which was not markedly different from that in previous studies.^3^, ^5^

The third patient profile identified in this study, eGFR ≥75 mL/min/1.73 m^2^ and treatment with an antithrombotic agent, was perhaps unexpected. To our knowledge, few studies have assessed the impact of antithrombotic agents on renal function. Diabetes mellitus is associated with hypercoagulability and platelet activation, which might contribute to renal microvasculature disorders.^36^ A recent study revealed that cilostazol attenuated the deterioration in albuminuria over 52 weeks in patients with type 2 diabetes^37^; therefore, it is possible that antithrombotic agents attenuate the decline in renal function by reducing thrombosis and vessel occlusion in the kidney. Further studies may be necessary to investigate this possibility.

We used the Q‐Finder algorithm,^28^ a supervised learning algorithm, to identify which patient profiles were more likely to show better RFP with SGLT2 inhibitors. After applying the Q‐Finder algorithm and reviewing the profiles, we found four profiles that showed class effects and class benefits favouring SGLT2 inhibitors over DPP‐4 inhibitors in terms of RFP. It is perhaps notable that three of the four profiles included eGFR as a factor, and suggest that patients with higher baseline eGFR (≥75 or ≥79 mL/min/1.73 m^2^) in combination with the second factor are more likely to experience RFP with an SGLT2 inhibitor than with a DPP‐4 inhibitor. Intriguingly, the change in HbA1c was similar between the two classes of drugs in each of the four profiles, suggesting that the benefit of SGLT2 inhibitors on RFP is associated with a similar change in glycaemic control relative to that observed with DPP‐4 inhibitors. These results should provide reassurance that the RFP benefit is accompanied by comparable glycaemic control in patients satisfying the criteria of these profiles.

The present study highlights the use of the Q‐Finder algorithm^28^ to identify patient subgroups likely to experience a clinical benefit from a specific treatment, in this case RFP, during treatment with an SGLT2 inhibitor versus a DPP‐4 inhibitor. We believe the approach used in this study is strengthened by combining a supervised learning algorithm for initial profile screening followed by expert review to evaluate the clinical relevance of the profiles.

It is important to consider the limitations of the present study, which include the smaller number of patients in the SGLT2 inhibitor group, which may have affected statistical power. It is also possible that the propensity score analysis may not fully account for (or exclude) any bias, given that there were marked differences in baseline characteristics, including general demographic characteristics and treatment history, between the two groups. Future studies of patients matched according to baseline characteristics/disease/treatment factors might be necessary to confirm the present findings. Further, as the MDV database comprises patients treated at large acute care hospitals in Japan, it may not be fully representative of patients treated in other clinical settings in Japan; therefore, the profiles obtained using the MDV database could be validated using other data sources. Because the creatinine value was measured up to 6 months before the index date, the baseline eGFR value included in the analysis may not reflect the patient's true baseline at the start of treatment. The primary outcome of the study was RFP, which was defined as no change or an increase in eGFR at 12 months (9‐15 months) after the index date. The study used a fixed follow‐up approach and required patients to continue treatment for at least 9 months. This could lead to selection bias because the patients included in the study are more compliant/persistent and are likely to have better outcomes than the general population of patients with type 2 diabetes. It is also possible that the observation period (9‐15 months) may have been too short to detect significant worsening of renal function in many patients. In addition, we cannot exclude possible washout or persisting effects of other prior or ongoing treatments. We also acknowledge that some patient characteristics and laboratory data were not entered by the hospital into the MDV database, resulting in some missing data. All diagnoses were identified using ICD‐10 codes. However, the claims data did not always include the actual diagnosis name, which possibly led to misclassification of diagnoses. Finally, we did not include administration of a number of therapeutic classes of drugs, such as non‐steroidal anti‐inflammatory drugs, which may themselves have nephrotoxic effects.

In conclusion, to our knowledge, this is the first study to investigate the profiles of patients who are likely to experience preservation of renal function during treatment with an SGLT2 inhibitor rather than with a DPP‐4 inhibitor in real‐world clinical practice. Our findings may help guide clinicians in their treatment decisions and identify which patients may benefit more from SGLT2 inhibitors. Our results also highlight the need to adjust for differences in baseline characteristics when comparing the effects of different classes of antidiabetic drugs in real‐world studies, particularly in large‐scale medical databases.

## CONFLICT OF INTEREST

F.L.Z. is an employee of Sanofi, USA. H.W. has received honoraria for scientific lectures from MSD K.K., Eli Lilly Japan K.K., Takeda Pharmaceutical Co., Ltd, Novartis Pharma K.K., Sumitomo Dainippon Pharma Co., Ltd, Sanofi K.K. and Daiichi Sankyo Company Ltd, and research funds from MSD K.K., Eli Lilly Japan K.K., Takeda Pharmaceutical Co., Ltd, Kowa Pharmaceutical Co., Ltd, Mochida Pharmaceutical Co. Ltd, Sanwa Kagaku Kenkyusho Co., Ltd, Novo Nordisk Pharma Ltd, Kissei Pharmaceutical Co., Ltd, Novartis Pharma K.K., Nippon Boehringer Ingelheim Co., Ltd, AstraZeneca K.K., Astellas Pharma Inc., Mitsubishi Tanabe Pharma Corporation, Sumitomo Dainippon Pharma Co., Ltd, Abbott Japan Co., Ltd, Sanofi K.K., Pfizer Japan Inc. and Daiichi Sankyo Company Ltd. Y.T. and Y.S. are employees of Sanofi K.K., Japan. M.B., D.Kang and C.E. are employees of Quinten, France. H.M. has received honoraria for scientific lectures from MSD K.K., Nippon Boehringer Ingelheim Co., Ltd, Astellas Pharma Inc., Ono Pharmaceutical Co., Ltd, Mitsubishi Tanabe Pharma Corporation, Sanofi K.K., Taisho Toyama Pharmaceutical Co., Ltd, Takeda Pharmaceutical Co., Ltd, Kowa Pharmaceutical Co., Ltd, Daiichi Sankyo Company Ltd, AstraZeneca K.K., Sanwa Kagaku Kenkyusho Co., Ltd, Novartis Pharma K.K. and Eli Lilly Japan K.K., research funds from Astellas Pharma Inc. and AstraZeneca K.K., and grants from Takeda Pharmaceutical Co., Ltd, Astellas Pharma Inc., MSD K.K., Teijin Pharma Ltd, Nippon Boehringer Ingelheim Co., Ltd, Kyowa Hakko Kirin Co., Ltd, Taisho Toyama Pharmaceutical Co., Ltd, Kowa Pharmaceutical Co., Ltd, Ono Pharmaceutical Co., Ltd, Daiichi Sankyo Company Ltd, Sanofi K.K., Mitsubishi Tanabe Pharma Corporation, Shionogi & Co., Ltd, Chugai Pharmaceutical Co., Ltd, Sunstar Inc., Otsuka Pharmaceutical Co., Ltd, Sanwa Kagaku Kenkyusho Co., Ltd, Sumitomo Dainippon Pharma Co., Ltd, Eisai Co., Ltd, Pfizer Japan Inc., Novo Nordisk Pharma Ltd, Mochida Pharmaceutical Co. Ltd, Novartis Pharma K.K. and Bristol‐Myers Squibb. D.Koya has received honoraria for scientific lectures from Astellas Pharma Inc., AstraZeneca K.K., MSD K.K., Ono Pharmaceutical Co., Ltd, Kyowa Hakko Kirin Co., Ltd, Taisho Toyama Pharmaceutical Co., Ltd, Taisho Pharmaceutical Co., Ltd, Takeda Pharmaceutical Co., Ltd, Mitsubishi Tanabe Pharma Corporation, Eli Lilly Japan K.K., Nippon Boehringer Ingelheim Co., Ltd, research funds from Sanwa Kagaku Kenkyusho Co., Ltd, Site Support Institute Co., Ltd, Mitsubishi Tanabe Pharma Corporation, and Morinaga & Co., Ltd, grants from Astellas Pharma Inc., AstraZeneca K.K., Ono Pharmaceutical Co., Ltd, Kissei Pharmaceutical Co., Ltd, Kyowa Hakko Kirin Co., Ltd, Kowa Pharmaceutical Co., Ltd, Sanofi K.K., Johnson & Johnson K.K., Daiichi Sankyo Company Ltd, Taisho Toyama Pharmaceutical Co., Ltd, Sumitomo Dainippon Pharma Co., Ltd, Takeda Pharmaceutical Co., Ltd, Mitsubishi Tanabe Pharma Corporation, Japan Tobacco Inc., Novo Nordisk Pharma Ltd, Bayer Yakuhin, Ltd and Pfizer Japan Inc., and courses endowed by Ono Pharmaceutical Co., Ltd, Kyowa Hakko Kirin Co., Ltd, Taisho Toyama Pharmaceutical Co., Ltd, Mitsubishi Tanabe Pharma Corporation and Nippon Boehringer Ingelheim Co., Ltd.

## AUTHOR CONTRIBUTIONS

F.L.Z and Y.T. were responsible for conceiving and/or designing the study. F.L.Z., Y.T., M.B., D. Kang. and C.E. were responsible for data acquisition and wrote the manuscript. H.W., Y.S., H.M. and D. Koya critically reviewed the manuscript. All authors contributed to data analysis or interpretation, gave final approval of the manuscript, and take accountability for the accuracy and integrity of the manuscript.

## Supporting information


**Appendix S1.** Supplemental methods: Patient eligibility

**Table S1.** ICD10 codes for diabetes mellitus
**Table S2.** Baseline characteristics
**Table S3.** Prior and concomitant antidiabetic therapy
**Table S4.** Comorbidities and medical history
**Table S5.** Medical therapies
**Table S6.** Baseline laboratory variables
**Table S7.** Proportions of patients overlapping multiple profiles
**Table S8.** Characteristics of the eight additional profiles: outcome = renal function preservation
