# Supplementary material for: Identification of subgroups of patients with type 2 diabetes with differences in renal function preservation, comparing patients receiving sodium‐glucose co‐transporter‐2 inhibitors with those receiving dipeptidyl peptidase‐4 inhibitors, using a supervised machine‐learning algorithm (PROFILE study): A retrospective analysis of a Japanese commercial medical database
Source: Diabetes Obes Metab. 2019 Jun 3;21(8):1925–34. doi: 10.1111/dom.13753 (PMC6771907; doi:10.1111/dom.13753)
Supplement: Supplementary file 1 — Appendix S1. Supplemental methods: Patient eligibility [file DOM-21-1925-s001.docx]

**Supplementary Appendix**

**Supplemental methods:** Patient eligibility

For the SGLT2 inhibitor group, patients were included if they were already using a DPP4 inhibitor prior to the index date, but patients who started taking a DPP4 inhibitor after the index date were excluded. For the DPP4 inhibitor group, patients who were taking any SGLT2 inhibitor before the index date were excluded.

Continuous treatment with an SGLT2 inhibitor or DPP4 inhibitor was defined as continuous treatment from the index date to the LOCF eGFR date in the follow-up period with a maximum treatment gap of 30 days. Patients who switched drugs within the same class were eligible, providing any treatment gap was <30 days. Patients with a treatment gap of ≤30 days were included if their LOCF eGFR was recorded ≤2 days after the projected end of the SGLT2 inhibitor prescription.

Six SGLT2 inhibitors (canagliflozin, dapagliflozin, empagliflozin, ipragliflozin, luseogliflozin and tofogliflozin) and nine DPP-4 inhibitors (once/twice-daily: alogliptin, anagliptin, linagliptin, saxagliptin, sitagliptin, teneligliptin and vildagliptin; once-weekly: trelagliptin and omarigliptin) were available in Japan at the time of the study. Treatments were identified based on Anatomical Therapeutic Chemical Classification System (ATC) code A10. Patients could use either class of drug in combination with other antidiabetic drugs or insulin. Patients in the SGLT2 inhibitor group could have used a concomitant DPP4 inhibitor if it was started before the index date.
